# Role of tryptophan residues of Erv1: Trp^95^ and Trp^183^ are important for its folding and oxidase function

**DOI:** 10.1042/BSR20150144

**Published:** 2015-08-28

**Authors:** Qi Wang, Swee Kim Ang, Efrain Ceh-Pavia, Jiayun Pang, Hui Lu

**Affiliations:** * Manchester Institute of Biotechnology, Faculty of Life Sciences, University of Manchester, 131 Princess Street, Manchester M1 7DN, U.K.; † Current address: Department of Microbiology, Yong Loo Lin School of Medicine, National University Health System, 5 Science Drive 2, National University of Singapore, Singapore 117597, Singapore; ‡ Department of Pharmaceutical, Chemical and Environmental Sciences, Faculty of Engineering and Science, University of Greenwich, Medway Campus, Central Avenue, Chatham Maritime, Kent ME4 4TB, U.K.

**Keywords:** flavin-adenine dinucleotide (FAD) binding, mitochondria, protein folding, tryptophan residue, thiol oxidase

## Abstract

Erv1 is an FAD-dependent thiol oxidase of the ERV (essential for respiration and viability)/ALR (augmenter of liver regeneration) sub-family and an essential component of the mitochondrial import and assembly pathway. Erv1 contains six tryptophan residues, which are all located in the highly conserved C-terminal FAD-binding domain. Though important structural roles were predicted for the invariable Trp^95^, no experimental study has been reported. In the present study, we investigated the structural and functional roles of individual tryptophan residues of Erv1. Six single tryptophan-to-phenylalanine yeast mutant strains were generated and their effects on cell viability were tested at various temperatures. Then, the mutants were purified from *Escherichia coli.* Their effects on folding, FAD-binding and Erv1 activity were characterized. Our results showed that Erv1^W95F^ has the strongest effect on the stability and function of Erv1 and followed by Erv1^W183F^. Erv1^W95F^ results in a decrease in the *T*_m_ of Erv1 by 23°C, a significant loss of the oxidase activity and thus causing cell growth defects at both 30°C and 37°C. Erv1^W183F^ induces changes in the oligomerization state of Erv1, along with a pronounced effect on the stability of Erv1 and its function at 37°C, whereas the other mutants had no clear effect on the function of Erv1 including the highly conserved Trp^157^ mutant. Finally, computational analysis indicates that Trp^95^ plays a key role in stabilizing the isoalloxazine ring to interact with Cys^133^. Taken together, the present study provided important insights into the molecular mechanism of how thiol oxidases use FAD in catalysing disulfide bond formation.

## Abstract (precis)

Erv1 (essential for respiration and viability 1) is a FAD-dependent sulphydryl oxidase with a tryptophan-rich catalytic domain. We show that Trp95 and Trp183 are important for stabilizing the folding, FAD-binding, and function of Erv1, whilst other four tryptophan residues are not functionally important.

## INTRODUCTION

Erv1 (essential for respiration and viability 1) is an essential component of the mitochondrial import and assembly (MIA) pathway, playing a critical role during import and oxidative folding of the mitochondrial intermembrane space (IMS) proteins [1–3]. In the MIA pathway, Mia40 acts as a thiol oxidoreductase and import receptor, interacting directly with the substrates of the pathway {e.g. Tim9 (translocase of inner membrane 9), Tim10, Cox17 (cytochrome oxidase 17) and Ccs1 (copper chaperone for superoxide dismutase 1)}. Erv1 is a FAD-dependent thiol oxidase belonging to the single domain ERV/ALR (augmenter of liver regeneration) oxidase family. It functions downstream of Mia40, catalysing re-oxidation of the reduced Mia40 and transferring electrons (via FAD) to molecular oxygen and/or cytochrome *c* [4–10].

**Figure 1:**
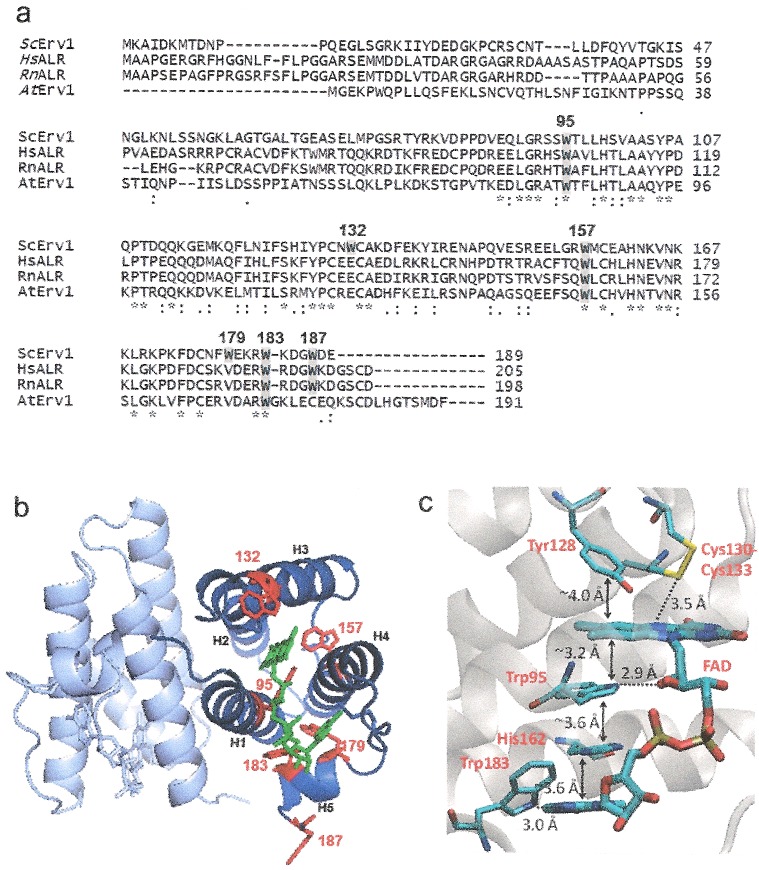
Cell viability of the Erv1 WT and tryptophan mutant strains (**a**) Sequence alignment of Erv/ALR proteins. *Sc*: *S. cerevisiae*, *Hs*: *Homo sapiens*, *Rn*: *Rattus norvegicus*, *At*: *Arabidopsis thaliana*. Tryptophan residues are highlighted in grey and numbered for yeast Erv1. (**b**) The X-ray crystal structure of yeast Erv1 core domain residues 84–188 with FAD and the six tryptophan residues shown (PDB code: 4E0OH) [11]. The structures were generated using the PyMOL VMD software. (**c**) Zoomed in structure of Erv1 showing how Trp^95^ is involved in both H-bonds and π-stacking interactions to stabilize FAD binding.

*Saccharomyces cerevisiae* Erv1 has a total of 189 amino acids including a highly conserved (among ERV/ALR family) FAD-binding domain of approximately 100 amino acids at the C-terminus (Figure 1). There are six conserved cysteine residues forming three pairs of disulfide bonds, with four of the cysteine residues located in the FAD-binding domain. Several structures of the FAD-binding domain of ERV/ALR proteins have been published [10–15], in which they are all crystallized as head-to-tail homodimers. Each subunit contains a four-helical bundle (H1–H4) that harbours the FAD cofactor and an additional (fifth) single-turn helix (Figure 1). The FAD-binding domain acts as a catalytic core (also called catalytic domain) containing a CXXC redox centre disulfide (Cys^130^–Cys^133^), located proximal to the isoalloxazine ring of FAD cofactor and a C-terminal CX_16_C structural disulfide. The shuttle disulfide (Cys^30^–Cys^33^) of Erv1 is located in the non-conserved N-terminal domain. It accepts the electrons from reduced Mia40 and transfers them to the active-site disulphide (C^130^-C^133^) and then in turn to the cofactor FAD and cytochrome *c* and/or molecular oxygen [4,16–19]. Erv1 and ALR employ a similar catalytic mechanism which involves several intermediate states, including a shuttle disulfide reduced state, S→FAD charge-transfer complexes and an FADH_2_-Erv1 state, during their catalytic cycle [17,20–22]. Whilst the shuttle and the active-site disulfides are functionally essential, the conserved CX_16_C disulfide (C^159^-C^176^) plays an important role in stabilizing the folding of Erv1 [6,23].

Tryptophan plays important roles in protein stability despite its scarcity in proteins. Erv1 has six tryptophan residues that are all located in the C-terminal FAD-binding domain making the domain tryptophan-rich. Among them Trp^95^, Trp^157^ and Trp^183^ are highly conserved across the ERV/ALR family; Trp^187^ is partially conserved; whereas Trp^132^ and Trp^179^ are not conserved (Figure 1a). Trp^95^, Trp^132^ and Trp^157^ are located in H1, H3 and H4 respectively; Trp^179^ and Trp^183^ are in the fifth single-turn helix (H5) and Trp^187^ is located at the very end of C-terminus, the third residue from the end (Figure 1a). Interestingly, the highly conserved Trp^183^ is next to the highly conserved residue Arg^182^, whose mutation in the human homologue ALR caused diseases. In 2009, Di Fonzo et al. [24] reported the first disease-associated mutant of ALR, in which a single conserved arginine to histidine substitution (ALR^R194H^) caused an autosomal recessive myopathy. Structural and functional study of the corresponding Erv1^R182H^ mutant showed that Arg^182^ plays an important role in the folding and FAD binding of Erv1 [25]. Although the oligomerization state of Erv1 *in vivo* is unclear, our recent *in vitro* study showed that whilst the purified full-length Erv1 formed a stable tetramer, the Erv1^R182H^ mutant was a homodimer under the same experimental condition [25]. Visualization of the structures of Erv1 [11] indicates that both Trp ^95^ and Trp^183^ side chains are involved in stabilizing the cofactor binding. Firstly, Trp^95^ forms an H-bond with the OH group of the ribitol moiety of FAD and Trp^183^ forms an H-bond with the nitrogen of adenine moiety of FAD using its side chain, suggesting a role similar to Arg^182^ in stabilizing FAD binding. Secondly, both Trp^95^ and Trp^183^ seem to play an important part in forming a series of π stackings with the cofactor FAD, involving the isoalloxazine ring of FAD, side chains of Trp^95^, Tyr^128^, His^162^, Phe^174^, Trp^183^ and the adenine ring of FAD (Figure 1c). In particular, the invariable Trp^95^ of ERV/ALR and Ero1 proteins locates in the centre of the aromatic stacking and is sandwiched between the isoalloxazine ring of FAD and the invariable His^162^ [11,13,26,27]. Though an important role can be assumed for some of the tryptophan residues, no experimental study on the importance of the tryptophan residues and the π stackings in ERV/ALR enzymes’ structure and function has been reported.

In the present study, we investigated the structural and functional roles of all six tryptophan residues of Erv1 with a focus on Trp^95^, using biochemical and biophysical methods, as well as computational and yeast genetic approaches. First, a set of six single tryptophan-to-phenylalanine yeast mutant strains were generated and their effects on cell viability were tested at various temperatures. Then, the tryptophan mutants were expressed and purified from *Escherichia coli.* Their effects on Erv1 folding, FAD-binding, stability and oxidase activity were studied using size exclusion chromatography, spectroscopic (absorption, CD and FAD fluorescence) and oxygen consumption analyses. Our results show that except Erv1^W183F^, all mutants were purified as a tetramer like the wild-type (WT) protein, whereas Erv1^W183F^ was a dimer. Erv1^W95F^ has the strongest effect on the stability and hence the function of Erv1, which is followed by Erv1^W183F^. Though the other mutants also result in observable effects on the folding and stability of Erv1, there is no obvious defect in the oxidase activity of the protein, including the highly conserved Trp^157^ mutant. Finally, computational analysis was performed to provide insight into the FAD-binding energy landscape at the atomic level, which assisted our understanding of the molecular mechanism by which Erv1^W95F^ causes the functional defect of Erv1.

## EXPERIMENTAL

### Site-directed mutagenesis

A construct encoding C-terminally LE(H)_6_-tagged full-length, WT Erv1 cloned into *E. coli* expression vector pET24a(+) (Novagen) using the Nde1 and Xho1 restriction sites was used as DNA template [28] to generate all mutant constructs for protein purification work in the present study. For yeast studies, *ERV1* together with endogenous promoter and terminator regions was inserted into the centromeric plasmid pRS414 as previously described [17] and was used as a DNA template for mutagenesis. Tryptophan-to-phenylalanine mutations were introduced by PCR using overlapping primer pairs containing the desired mutation point. Plasmid DNA constructs with the correct mutation was verified by DNA sequencing.

### Protein purification

WT Erv1 and tryptophan mutants were expressed in *E. coli* strain Rosetta-gami™ 2 (Novagen) and purified as previously described [17,25]. Briefly, protein was expressed in the presence of 0.5 mM IPTG and 10 μM FAD at 16 °C for 16–20 h. Induced cell pellets resuspended in buffer A (150 mM NaCl, 50 mM Tris/HCl, pH 7.4) containing 5 mM imidazole, 50 μM FAD and one tablet of EDTA-free protease inhibitor cocktail (Roche) were lysed by sonication on ice. The supernatant fraction containing LE(H)_6_-tagged Erv1 was bound to 2–3 ml of Ni^2+^-charged histidine. Bind resin (Novagen) pre-equilibrated with binding buffer. Following a washing step with 20–30 ml of wash buffer (buffer A plus 20 mM imidazole), the protein was eluted with 4–6 ml of elution buffer (buffer A containing 500 mM imidazole). FAD (100 μM) was added to eluted proteins before storage at −80°C until further use. The affinity-purified proteins were further separated for use in *in vitro* studies by gel filtration chromatography using a Superdex 200 (or Superdex 75) 10/30 column connected to an ÄKTA-FPLC system (GE Healthcare) at 4°C in BAE (150 mM NaCl, 50 mM Tris/HCl, 1 mM EDTA, pH 7.4).

### UV-visible spectroscopy

Absorption spectra were recorded from 250 to 700 nm, at 1 nm intervals, in a 1 cm path-length quartz cuvette using a Cary 300 Bio UV-Visible spectrophotometer (Varian Ltd.). Measurement of the molar extinction coefficient was done in BAE as previously described [6,7]. The calculated molar extinction coefficients are summarized in Table 1. The FAD content of the WT and mutant Erv1 was calculated based on the eqn (1), an extinction coefficient for free FAD (fFAD) of 11.3 mM^−1^ cm^−1^ and the absorption spectrum of Erv1 or mutants in BAE plus 1% SDS.

1 $$FAD\%=\frac{{ɛ}_{280}\;\times \;A_{450}}{11.3\;\times \;(A_{280}-1.82\;\times \;A_{450})}$$

**Table 1:** Summary of the properties of FAD-binding and stability of the WT and mutant Erv1 The parameters were determined in BAE at pH 7.4 under the same conditions for all proteins as described in the ‘Experimental’ section. ^a.^ T_m_ determined based on FAD fluorescence; ^b.^ T_m_ determined based on CD at 222 nm. Errors represent means ± SE, n≥2.

Erv1	*λ*_max_ (nm)	*ε* (mM^−1^cm^−1^)	FAD%	*T*_m_ (°C)*	*T*_m_ (°C)^†^
WT	460±1	12.3	95±3	64±2	66±2
W95F	458±2	11.8	93±5	42±2	45±3
W132F	460±1	12.2	94±3	64±2	65±2
W157F	460±1	12.3	92±3	54±2	55±2
W179F	460±1	12.3	100±3	57±2	56±2
W183F	459±1	11.6	71±3	51±2	50±2
W187F	461±1	11.7	94±3	59±2	58±2

*ε*_280_ is the extinction coefficient of the apoprotein; 42.54 mM^−1^ cm^−1^for the WT and 36.85 mM^−1^ cm^−1^ for the single tryptophan-to-phenylalanine mutants of Erv1. *A*_280_ and *A*_450_ are absorbance at 280 and 450 nm respectively. The ratio of *ε*_280_/*ε*_450_ of fFAD in BAE plus 1% SDS determined in the present study is 1.82.

### CD

CD analysis was done using a Chirascan CD spectrometer (Applied Photophysics Ltd.) and either a 1 mm (far UV) or 5 mm (near UV) path-length quartz cuvette. Each spectrum represents an average of four independent scans with the spectra for buffer alone subtracted. Thermal denaturation was measured at 222 nm in 1°C intervals over 5°C–90°C, with a temperature increase of 1°C/min. Melting temperatures (*T*_m_) were calculated by obtaining the first derivative of each profile and finding the maximum.

### Fluorescence spectroscopy

Fluorescence measurements were recorded using a Cary Eclipse fluorescence spectrophotometer (Varian Ltd.) in a 1 cm × 0.2 cm path-length quartz cuvette. Thermal denaturation was followed by the increase in FAD fluorescence at 535 nm after excitation at 455 nm, in 1°C intervals between 5°C–90°C, with a temperature increase of 1°C/min.

### Oxygen consumption assays

Erv1 oxidase activity was measured using a Clark-type oxygen electrode (Hansatech Instrument Ltd) in a 0.5 ml of reaction volume at 25°C or 37°C in BAE as previously described [6]. Data analysis of the oxygen consumption profile and the calculation of the reaction slope were performed using the Microcal™ Origin™ statistical software package.

### Yeast complementation assays

A ∆*erv1* knockout strain containing WT *ERV1* in a *URA3* plasmid [24] was co-transformed with WT or mutant *ERV1* on *TRP1* plasmid pRS414. For counter-selection tests, the strains were spotted on 5-fluoroorotic acid (FOA) plates and incubated for up to 2–3 days at 25°C, 30°C or 37°C.

### Miscellaneous

All experiments were carried out in BAE (150 mM NaCl, 50 mM Tris/HCl, 1 mM EDTA, pH 7.4) unless specifically stated. For multi-angle laser light-scattering, protein samples were applied to a Superdex 200 10/30 gel filtration column (GE Healthcare) running with buffer A. Proteins eluting from the column passed through an in-line DAWN EOS laser photometer set at 682 nm and an Optilab rEX refractor. To calculate the weight-averaged molecular mass, the light scattering intensity and eluent refractive index were analysed using ASTRA version 4.8 software.

### MD simulations

MD simulations of the WT and Erv1^W95F^ mutant were performed using AMBER12 [29] with the protein modelled by the AMBER ff99SB force field [30] and the substrate FAD represented using the parameters and partial charges developed by Asada et al. [31]. The initial protein co-ordinate was taken from the crystal structure of the C-terminal core domain (CTD) of Erv1 from *Saccharomyces cerevisiae* at 2.0 Å (1 Å=0.1 nm) resolution (PDB code: 4E0H) [11]. In the crystal structure, the CTD of Erv1 exists as a homodimer, with each subunit formed of a four-helix bundle that accommodates FAD binding. All crystallographic waters were removed whereas hydrogens were added using the LEAP program within AMBER12. The protein–FAD complex consisting of 206 amino acid residues (103 residues in each subunit) and two FADs was solvated in a rectangular TIP3P water box with at least 8 Å between the edge of the box and the protein. Chloride ions were added to neutralize the overall change of the system. Following minimization, the system was heated to 298 K, equilibrated for 200 ps under the constant pressure condition and the production simulation was performed for 1 ns.

### Calculation of binding free energy

The MM-GBSA approach [32,33] implemented in AMBER12 was used to study the FAD binding energy to Erv1 WT (Δ*E*_WT_) and Erv1^W95F^ mutant (Δ*E*_W95F_). A total of 500 frames from the 1-ns MD trajectory were used. The MM–GBSA free energy was obtained from a combination of the gas phase molecular mechanics energy term (*E*_MM_=*E*_ele_ + *E*_vdw_ + *E*_int_) and the solvation free energy term (*G*_solvation_=*G*_polar_ + *G*_nonpolar_) without the solute entropy term. The difference in the energy of FAD binding in the WT and W95F was derived by ΔΔ*E*=Δ*E*_W95F_ − Δ*E*_WT_.

## RESULTS

### Effects of Erv1 tryptophan mutations on yeast cell viability

**Figure 2:**
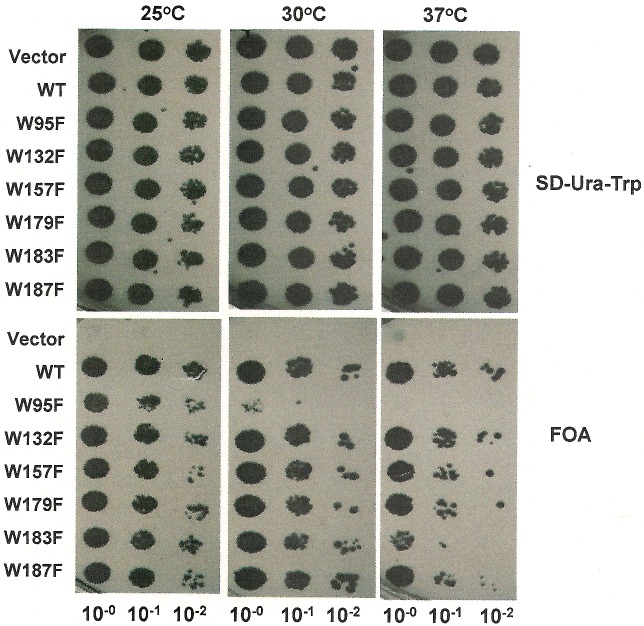
Sequence alignment of ERV/ALR homologues and structure of Erv1 Yeast complementation assays with tryptophan mutant Erv1 expression via plasmid shuffling in the absence and presence of FOA. Strains containing empty vector or Erv1 WT were used as negative and positive controls respectively. Cells were grown at 25°C, 30°C or 37°C for 2–3 days.

To investigate the requirement of individual tryptophan residues of Erv1 in its function *in vivo*, we generated six tryptophan mutant yeast strains by expressing plasmid-encoded *erv1* single tryptophan-to-phenylalanine mutants, in an *ERV1* knockout strain ∆*erv1*, isogenic to the WT Trp^303^, as described previously [24]. Then, effects of the mutation on the cell viability were checked by spot-testing at 25°C, 30°C and 37°C (Figure 2). Upon counter-selection of WT *ERV1* (on *URA3*-containing plasmid), the Erv1^W95F^ mutant was temperature sensitive at both 30°C and 37°C, Erv1^W183F^ grew normally at 25°C and 30°C, but slowly at 37°C, whereas the other four mutants showed no obvious growth defect at all three temperatures. This result confirmed that Trp^95^ and to a lesser extent Trp^183^ play important roles in the function of Erv1 *in vivo*. The other four tryptophan residues, including the highly conserved Trp^157^, were not important for cell viability under these experimental conditions.

### Effects of tryptophan mutations on the folding and FAD-binding of Erv1

**Figure 3:**
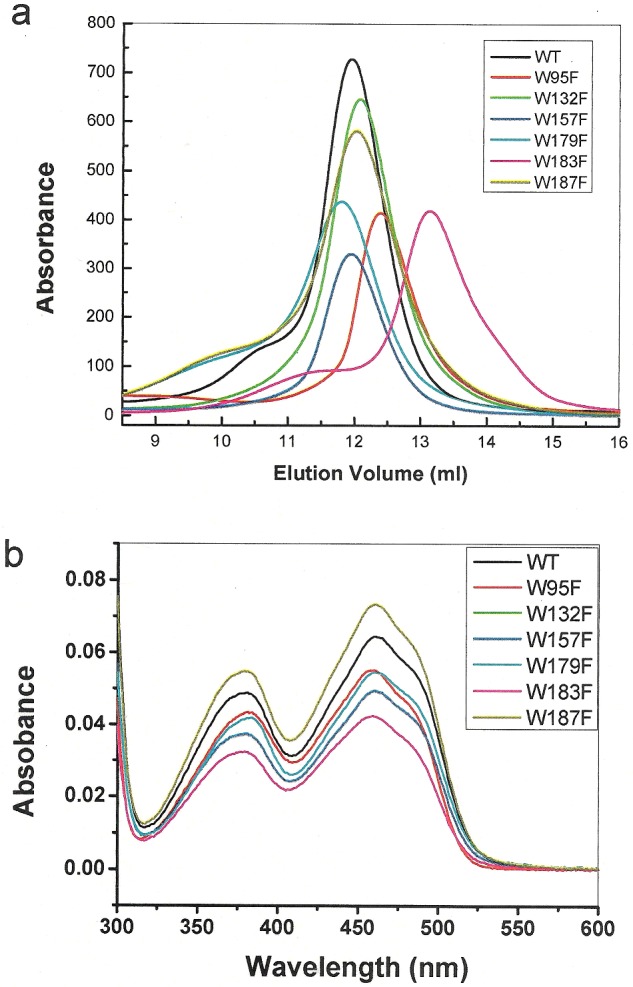
Oligomerization state and absorption spectra of the WT and tryptophan mutants of Erv1 (**a**) Gel filtration chromatography profiles of the WT (black line) and six tryptphan mutants (as indicated) on a Superdex 200 column. (**b**) UV-visible spectra of the WT and tryptophan mutants.

To understand the structural and functional roles of tryptophan residues of Erv1, six single tryptophan-to-phenylalanine mutant constructs of Erv1 were generated and expressed in *E. coli.* All mutants were successfully purified using the same method as that used for the WT protein, as described previously [6,17]. Gel filtration chromatography analysis showed that apart from Erv1^W183F^ all other five mutants eluted in the same or similar position as the WT protein and thus existed mainly in a tetramer form like the WT Erv1 [17,25] (Figure 3a; Supplementary Figure S1a). Elution of Erv1^W183F^ was delayed and the molecular mass of Erv1^W183F^ was determined using light scattering analysis to be 44±3 kDa (Supplementary Figure S1b), suggesting that Erv1^W183F^ formed a dimer (monomer Erv1 is 22 kDa).

Though there is a difference in oligomerization state, all six mutants displayed the same yellowish colour as the WT protein and showed very similar overall UV-vis absorption spectra profiles (Figure 3b). The visible absorption maximum *λ*_max_ was shifted most by Erv1^W95F^, from 460 nm (for WT) to 458 nm (Table 1). The result suggests that FAD cofactor binding was not significantly affected by the mutation. Consistently, similar extinction coefficients were obtained as shown in Table 1, which were determined by adding of 1% SDS to release FAD as described previously [6,7]. Moreover, FAD content of each purified mutant was determined (see methods). Apart from Erv1^W183F^ all the other five mutants had ∼95% FAD content, very similar compared with the WT Erv1 and thus one FAD per monomer protein. However, the FAD content of Erv1^W183F^ decreased by more than 20%, indicating that Trp^183^ and possibly also the tetramer formation play a role in stabilizing cofactor binding.

**Figure 4:**
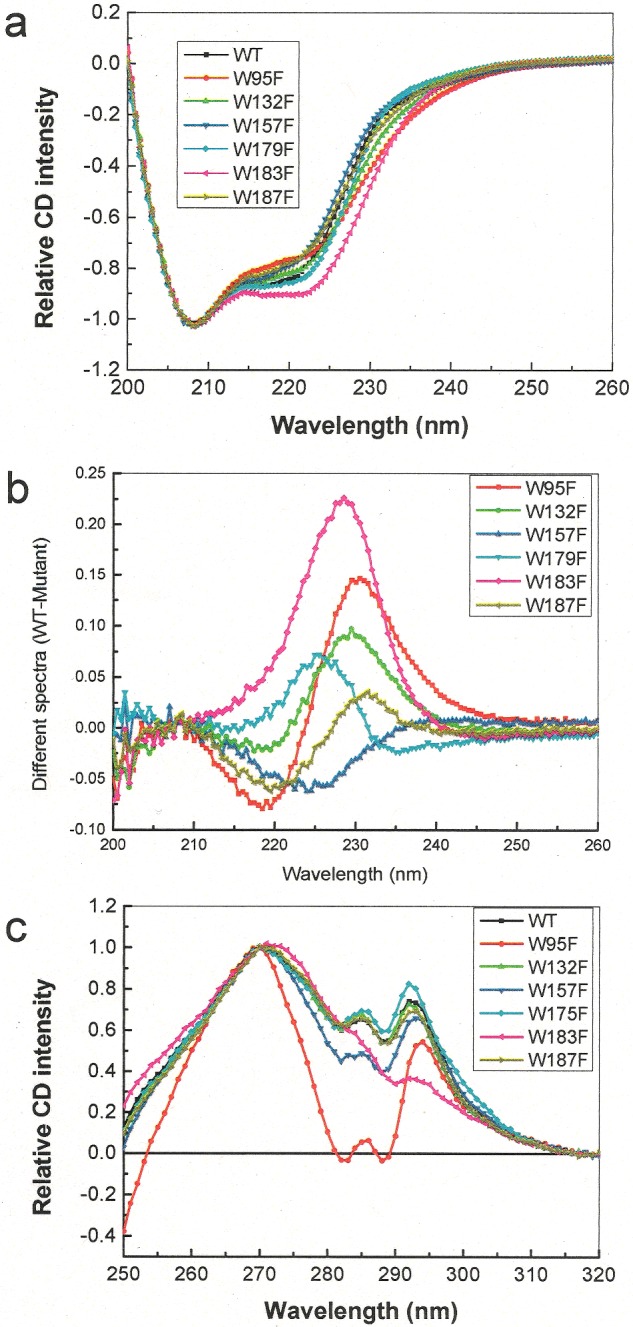
CD spectra of the WT and Trp mutants of Erv1 (**a**) Far UV CD spectra of 10 μM the WT (black line) and tryptophan mutants. (**b**) Different spectra of the far UV CD spectra obtained by subtraction of the mutant CD spectrum from that of the WT in (**a**). (**c**) Near UV CD spectra of the WT and tryptophan mutants of Erv1. All the colours were used systematically as in Figure 1.

Next, far and near UV CD analyses were carried out to investigate the effects of the tryptophan mutants on the folding of Erv1. The spectral profiles were compared with that of the WT protein in relative intensity since they were different in their FAD content and thus holoprotein concentration. The far UV CD spectra (Figure 4a) were normalized at 208 nm. Overall, all the spectra showed characteristics of highly α-helical conformations, but with some differences in the region from 215 to 240 nm, especially for Erv1^W95F^ and Erv1^W183F^. Subtraction of the Erv1^W183F^ spectrum from that of the WT gave a bell-shaped peak centred at 230 nm, characteristic of strong aromatic–aromatic interactions (Figure 4b). In terms of the differences between the WT and Erv1^W95F^ spectra, apart from a positive peak at ∼230 nm, there was also a negative peak at ∼220 nm suggesting loss of some α-helical structure in Erv1^W95F^. Similarly, clear differences in the near UV CD spectra between the WT and these two mutants (Erv1^W95F^ and Erv1^W183F^) were observed (Figure 4c). The result showed that the peak of Erv1 near UV CD at 285 nm was contributed mainly by Trp^95^, whereas Trp^183^ seemed to contribute to the peak at 295 nm significantly. Furthermore, the facts that the near UV CD spectrum of Erv1^W183F^ was closely similar to that of Erv1^R182H^ and that they both formed a dimer (rather than tetramer) indicated that the signal at 295 nm may be associated with formation of an Erv1tetramer.

Taken together, these results showed that both Trp^95^ and Trp^183^ are structurally important in folding and FAD binding of Erv1. Whereas Erv1^W95F^ affected the secondary and tertiary structure of Erv1 without affecting its oligomerization state, Erv1^W183F^ disrupted not only the oligomeric state, but also the secondary structure and tertiary aromatic–aromatic interactions and decreased FAD content under the experimental conditions.

### Effects of the tryptophan mutations on the stability of Erv1

**Figure 5:**
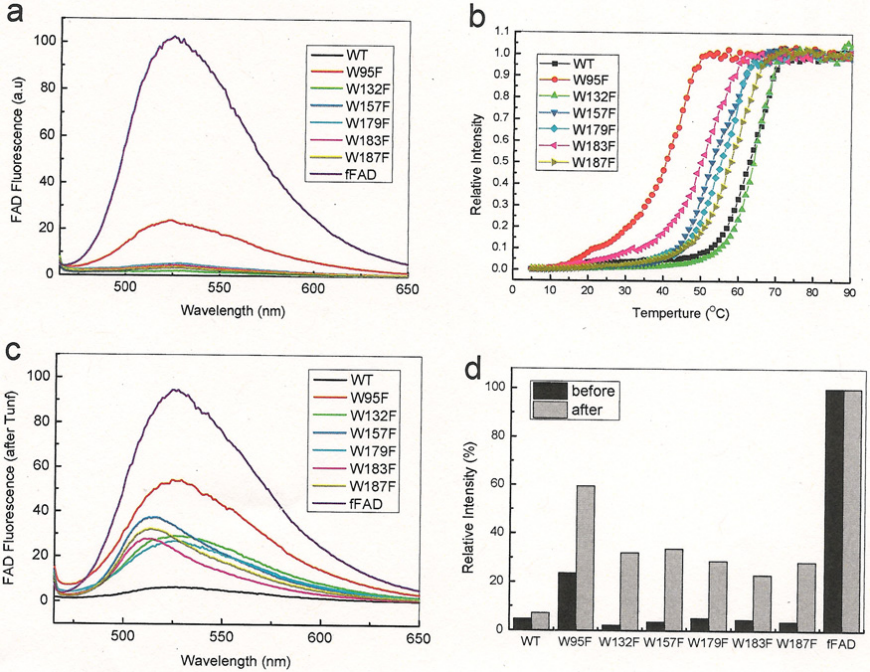
FAD fluorescence spectra and thermal stability of the WT and tryptophan mutants of Erv1 (**a** and **c**) FAD fluorescence spectra (10 μM) of the WT, tryptophan mutants and fFAD measured at 5°C before (**a**) and after (**c**) thermal denaturation as shown in (**b**). (**b**) Thermal denaturation of the WT and mutant proteins followed by FAD fluorescence with excitation at 450 nm and emission at 527 nm. (**d**). Relative total FAD fluorescence intensity (450–650 nm) of the proteins before (black) and after (grey) thermal denaturation normalized to that of fFAD as 100%.

It has been shown that FAD release and thermal unfolding of Erv1 are a co-operative process [25]. Thus, we investigated how each tryptophan mutation affects the thermal stability of the protein based on FAD fluorescence intensity change. Firstly, FAD fluorescence spectra of the WT and six mutants and the same concentration of fFAD were recorded at 5°C. As shown in Figure 5(a), whereas W95F displayed ∼20% fFAD intensity, negligible fluorescence intensity was detected for the WT and other mutants due to Erv1 binding quenching FAD fluorescence. Secondly, temperature-dependence of FAD fluorescence intensity change and thus the cofactor release was measured (Figure 5b) and the results were summarized in Table 1. Apart from W132F, which showed the same midpoints of thermal denaturation (*T*_m_) of 64°C as the WT Erv1, all other five mutants displayed decreased thermal stability. W95F is the most unstable mutant with a *T*_m_ of 41°C, which is 23°C lower than that of the WT protein. For W183F, *T*_m_ of 51°C, a decrease of ∼13°C was observed (Table 1). W95F had the strongest effect on the stability of Erv1, followed by W183F, W157F and the non-conserved tryptophan mutants W179F, W187F, whereas W132F showed no obvious effect on the thermal stability of Erv1 (Figure 5b; Table 1). This order is not surprising, as the first three residues (W95F, W183F and W157F) are highly conserved whereas the last three are not (Figure 1). Similar *T*_m_ values were determined for the mutants based on CD intensity change at 222 nm (Table 1), showing that Erv1 unfolding and cofactor release was a coupled process.

To gain more understanding of the reversibility of the cofactor binding, the FAD fluorescence spectra of the proteins after thermal denaturation were re-recorded at 5°C (Figure 5c). There was little change to WT Erv1 and fFAD following renaturation. However, clear FAD fluorescence intensity increases were observed for all six mutants (Figures 5c and 5d), showing that there was a defect in re-folding and/or cofactor binding in the mutants after thermal denaturation. In summary, consistent with the conclusion above, temperature-dependent studies showed Erv1^W95F^ and Erv1^W183F^ had the strongest effect on the stability of Erv1 and FAD-binding, though all six mutants displayed decreased thermal stability of Erv1.

### Effects of tryptophan mutations on Erv1 oxidase activity

**Figure 6:**
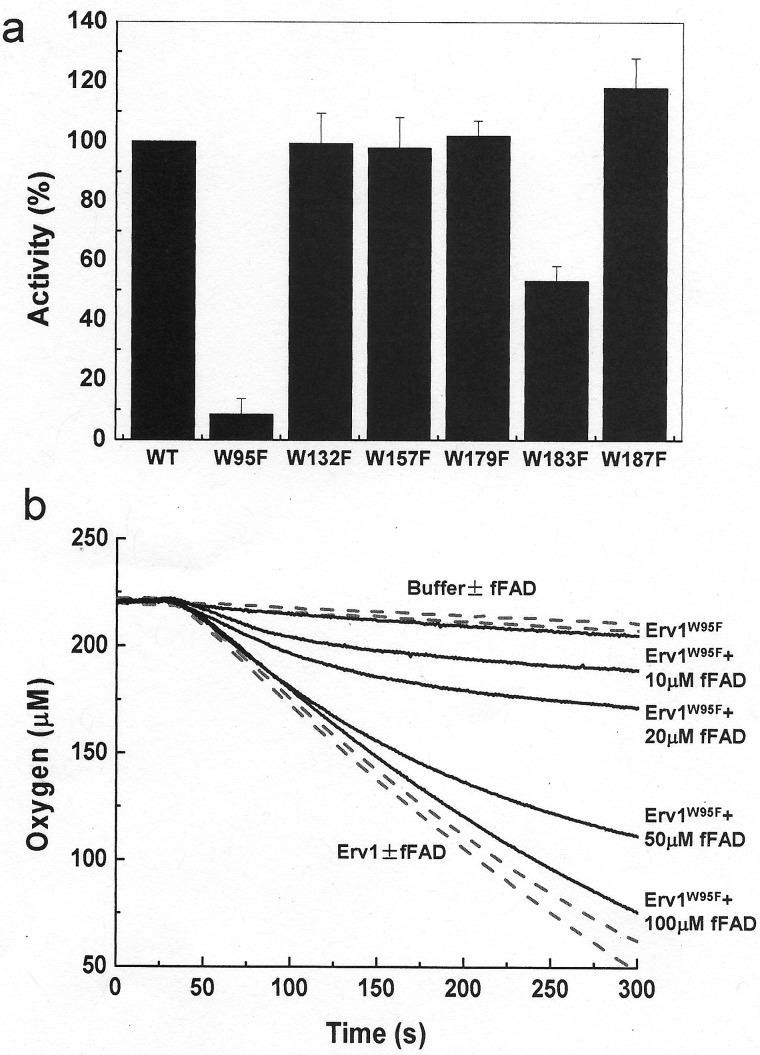
Oxidase activity of the WT and tryptophan mutants of Erv1 (**a**) The relative activities of the WT and six tryptophan mutants of Erv1 measured based on oxygen consumption at 37°C. The activities were normalized to that of the WT Erv1 as 100%. All reactions contained 3 μM of the WT or mutant protein, using 5 mM TCEP as the electron donor in the presence of SOD (superoxide dismutase). The initial rates of oxygen consumption were used to calculate the relative activities. Errors represent means ± S.E.M., *n* ≥ 3. (**b**) Time courses of oxygen consumption at 37°C catalysed by 1 μM of WT with/without addition of 100 μM fFAD or 1 μM W95F mutant in the presence of various fFAD concentrations as indicated in the figure.

To understand the importance of the tryptophan residues in Erv1 function, the oxidase activity of the WT and mutants was analysed using oxygen consumption assay with 5 mM TCEP [tris-(2-carboxyethyl) phosphine] as substrate as established previously [25]. The relative activity of each mutant was calculated based on their initial oxygen consumption rate compared with that of the WT Erv1 (Table 2; Figure 6a). At 25°C and 30°C, only Erv1^W95F^ showed a clear functional defect, with ∼50% activity of the WT Erv1 (Table 2). At 37°C, both Erv1^W95F^ and Erv1^W183F^ displayed a significant functional defect; which Erv1^W95F^ showed ∼9% and Erv1^W183F^ had ∼53% activity of the WT Erv1 respectively (Figure 6a). Under the same conditions, the other four mutants, including the highly conserved Trp^157^ mutant, showed no obvious functional defect and Erv1^W187F^ was slightly more active than the WT Erv1 at all three temperatures. These results are consistent with our *in vivo* findings and provide a good explanation for the temperature sensitive growth phenotypes we observed (Figure 2).

**Table 2:** Oxidase activity and cell viability of the WT and mutant Erv1 The activities were determined based on the initial oxygen consumption rate using that of the WT as 100%. Oxygen consumption catalysed by 3 μM of the WT or a tryptophan mutant using 5 mM TCEP as the electron donor in the presence of SOD as described in the ‘Experimental’ section. Errors represent means ± S.E.M., *n* ≥ 3. The cell growth phenotypes were determined based on spot-test shown in Figure 7. Abbreviations: SOD, superoxide dismutase; ts, temperature sensitive.

Erv1	Activity at 25°C	Activity at 30°C	Activity at 37°C	Cell viability
WT	100	100	100	WT
W95F	53±7	46±5	9±5	ts at 30°C and 37°C
W132F	90±8	107±5	99±2	WT-like
W157F	84±4	109±12	98±2	WT-like
W179F	95±4	99±5	102±5	WT-like
W183F	102±7	112±5	53±5	ts at 37°C
W187F	108±2	128±5	118±5	WT-like

Our previous study showed that the Erv1^R182H^, a disease related mutant, has stronger effects on folding and FAD-binding of the catalytic reaction intermediates of Erv1 than the oxidized steady state enzyme [25]. The cofactor was released readily from Erv1^R182H^ during its catalytic cycle leading to complete inactivation of the enzyme. In the present study, although only ∼30% of the oxidized Erv1^W95F^ and 10% of Erv1^W183F^ were denatured at 37°C (Figure 5b), the activity loss was higher, ∼90% and 50% respectively. Thus, we reasoned that similar to Erv1^R182H^, Erv1^W95F^ and Erv1^W183F^ had stronger destabilization effects on folding and FAD-binding of the reaction intermediates than on the initial oxidized Erv1. To test this hypothesis, the oxygen consumption experiment was carried out in the presence of fFAD. As expected, the oxidase activities of these mutants were recovered by addition of fFAD and in a fFAD concentration dependent manner (Figure 6b). Moreover, no obvious differences in the stability of the oxidized enzymes were observed based on thermal denaturation studies. *T*_m_ of 66±1°C for the WT and 46±2°C for the W95F mutant were obtained in the presence of 10 μM fFAD, which are the same as those determined in the absence of fFAD (Table 1).

### Computational analysis

**Figure 7:**
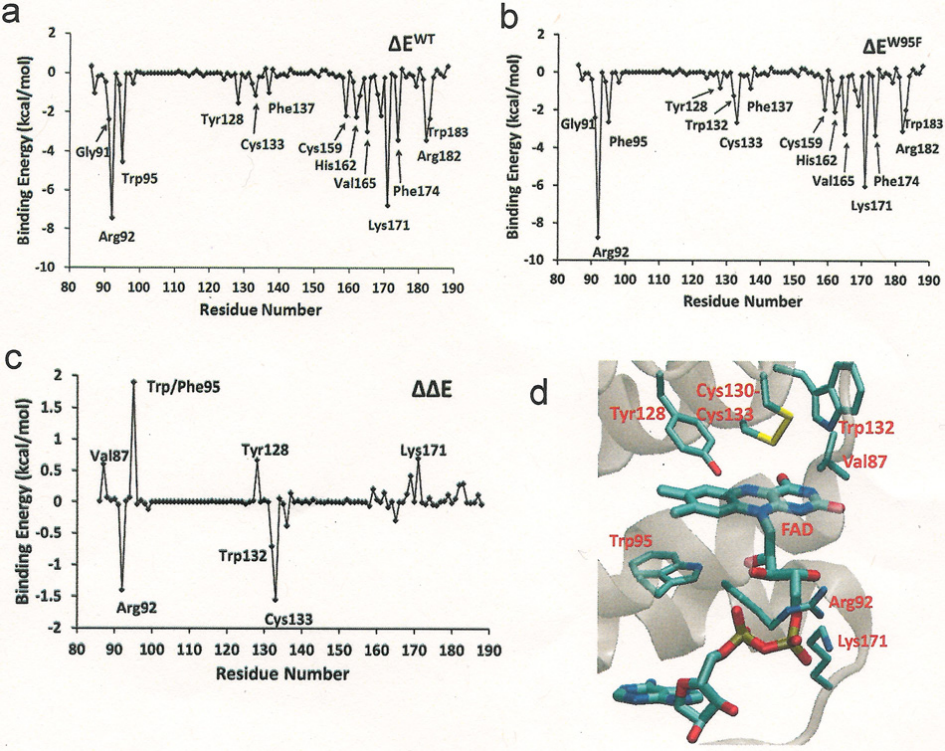
Computational analysis (**a**) Decomposition of the total FAD-binding energy into the contribution of individual residues for the WT Erv1 and (**b**) the decomposition plot for Erv1^W95F^. (**c**) Changes in the energy of FAD binding by individual residues in the WT and W95F (ΔΔE=ΔE^W95F^–ΔE^WT^). Residues with ΔΔE greater than ±0.5 kcal/mol are labelled. (**d**) Zoomed in structure of Erv1 showing residues whose binding energies to FAD are perturbed the most (|ΔΔE| > 0.5 kcal/mol) by the W95F mutation as shown in (**c**).

To understand the effects of W95F mutant on the stability of FAD-binding further, MD simulations (See ‘Experimental’) of the WT and Erv1^W95F^ mutant were performed based on the crystal structure of the CTD of Erv1 at 2.0 Å resolution (PDB code: 4E0H) [11]. The overall FAD-binding energies of the WT Erv1 [−71.1±6.0 kcal/mol (1 cal ≡ 4.184 J)] and Erv1^W95F^ (−73.7±4.7 kcal/mol), as calculated by the MM-GBSA method, are similar. When the binding energy is decomposed into the contribution of individual residues, key residues that contribute to FAD-binding are identified as shown in Figures 7(a) and 7(b). The residues include Gly^91^, Arg^92^, Trp^95^, Tyr^128^, Cys^133^, Phe^137^, Cys^159^, His^162^, Val^165^, Lys^171^, Phe^174^, Arg^182^ and Trp^183^. Consistently, these key residues are all located in proximity to and interacting with the isoalloxazine ring, ribitol moiety and adenosine moiety of FAD respectively (Supplementary Figure S1). However, apparent differences in the contribution of individual residues between the WT and the W95F mutant were observed. The residues with significant binding energy changes (ΔΔE > ±0.5 kcal/mol) are Val^87^, Arg^92^, Trp/Phe^95^, Tyr^128^, Trp^132^, Cys^133^ and Lys^171^ (Figure 7c) and interestingly they are all positioned in close proximity to the isoalloxazine ring of FAD (Figure 7d). The result indicates that Trp^95^ plays a key role in stabilizing the isoalloxazine ring in its positioning within the binding site and how it interacts with key residues, such as Cys^133^ for which the binding energy to FAD is changed by 1.55 kcal/mol in Erv1^W95F^ (Figure 7c).

## DISCUSSION

In the present report, we investigated the structural and functional roles of all six tryptophan residues of Erv1, using yeast genetic, biochemical and computational methods, with focus on the highly conserved Trp^95^. We showed that all the tryptophan mutants apart from W132F, had an impact on the thermal stability of Erv1 as evidenced by their decreased Tm (Figure 5; Table 1). In terms of function, the three non-conserved tryptophan residues (Trp^132^, Trp^179^ and Trp^187^) and Trp^157^, a highly conserved residue in the ERV/ALR sub-family, seem not to be essential for Erv1 function under normal cell growth or our experimental conditions (Figure 1; Table 2). However, both Trp^95^ and Trp^183^ play an important role in stabilizing the folding and FAD-binding and thus the function of Erv1.

Mutagenesis studies of several FAD/FMN-dependent enzymes suggested that aromatic residues forming π-stacking interactions with the isoalloxazine ring of FAD/FMN help stabilize formation of the anionic flavin hydroquinone intermediate [34–36]. Similarly, in our study, mutating Trp^95^ into phenylalanine affects the π–π repulsive interactions between the ring systems, which may modulate the catalytic steps for the formation of the S→FAD charge–transfer complexes. Our experimental results showed that Erv1^W95F^ has the strongest effect on the stability and oxidase activity of Erv1. The functional defect of Erv1^W95F^ is largely due to the destabilizing effects on the catalytic reaction intermediates since its activity can be recovered and sustained by addition of fFAD in the reaction. This observation is consistent with the result of our computational analysis, suggesting that Trp^95^ is crucial in stabilizing the isoalloxazine ring to interact with Cys^133^, hence the redox centre disulfide. Consequently, Erv1^W95F^ mutation causes a strong functional defect of Erv1 and impaired cell growth at both 30°C and 37°C.

Trp^183^, on the other hand, is not directly involved in the π-stacking network and only forms an H-bond with the nitrogen of adenine moiety of FAD. This serves to maintain the orientation of the adenine ring (FAD is in a bent conformation with the isoalloxazine and the adenine ring buried in the active site and sandwiched between Trp^95^ and His^162^). Therefore, mutating Trp^183^ to phenylalanine is not as severe as W95F in terms of its effect on Erv1 oxidase function (Table 2). However, Erv1^W183F^ showed a significant effect on the oligomerization state of Erv1, which leads to a clear functional and growth defect at 37°C but not at 30°C. This result is the same as that caused by Erv1^R182H^, a disease related mutant, forming a dimer under the same experimental conditions [25]. The fact that both Arg^182^ and Trp^183^ are part of the fifth single turn α-helix (H5) of Erv1 shows that this region is important in stabilizing the tetramer formation. Furthermore, Arg^182^ may form intermolecular hydrogen bonds to stabilize the tetramer formation, which may be assisted by a typical side-chain cation–π interaction with Trp^183^. Since tryptophan is more favoured to interact with cationic side chains than phenylalanine [37], we hypothesize that mutation of Trp^183^ disrupts the cation–π interaction with Arg^182^, causing disruption of the intermolecular interactions mediated by Arg^182^ and subsequent dissociation of the tetrameric structure of Erv1. Such a molecular mechanism of oligomerization formation has been observed previously [38]. Together with the observation that purified W183F mutant showed a decreased FAD content than the WT and other tryptophan mutants (Table 1), our results suggest that tetramer formation plays an important role in stabilizing the cofactor binding by Erv1 through the cation–π interaction between Arg^182^ and Trp^183^ and Trp^183^’s contribution in maintaining the π–π stacking network.

More generally, the aromatic ring stacking is conserved not only in ERV/ALR family, but all FAD-dependent thiol oxidases, including Ero1 (a thiol oxidase of the endoplasmic reticulum) that has no sequence homology with ERV/ALR enzymes [26,27,39]. Thus, it is tenable that the extensive π–π stacking around FAD is the key to create a rigid active site to receive electrons from the active-site disulfide (Cys^130^–Cys^133^) and pass electrons to molecular oxygen. Taken together, the current study contributes to our understanding of how thiol oxidases use FAD to catalyse the disulfide bond formation and broadly, provide insights into the function and mechanism of the structurally diverse flavinenzymes.

## Abbreviations

ALR: augmenter of liver regeneration
Erv: essential for respiration and viability
fFAD: free FAD
FOA: 5-fluoroorotic acid
MIA: mitochondrial import and assembly
TCEP: tris-(2-carboxyethyl) phosphine
WT: wild-type
